# Prevalence, Determinants, and Prognostic Significance of Hospital Acquired Pneumonia in Patients with Acute Heart Failure

**DOI:** 10.3390/jcm9072219

**Published:** 2020-07-13

**Authors:** Atsushi Tada, Kazunori Omote, Toshiyuki Nagai, Yasuyuki Honda, Hiroki Nakano, Satoshi Honda, Naotsugu Iwakami, Yasuhiro Hamatani, Michikazu Nakai, Kunihiro Nishimura, Yasuhide Asaumi, Takeshi Aiba, Teruo Noguchi, Kengo Kusano, Hiroyuki Yokoyama, Satoshi Yasuda, Hisao Ogawa, Toshihisa Anzai

**Affiliations:** 1 Department of Cardiovascular Medicine, Faculty of Medicine and Graduate School of Medicine, Hokkaido University, Sapporo 0608638, Japan; atsushi-tada.0409@med.hokudai.ac.jp (A.T.); komote@med.hokudai.ac.jp (K.O.); anzai@med.hokudai.ac.jp (T.A.); 2 Department of Cardiovascular Medicine, National Cerebral and Cardiovascular Center, Osaka 5648565, Japan; hondayasuyuki.for.office@gmail.com (Y.H.); hiroki08192001@gmail.com (H.N.); apeqtec@yahoo.co.jp (S.H.); naoi1115@gmail.com (N.I.); y.hamatani1114@gmail.com (Y.H.); asaumiyasu@yahoo.co.jp (Y.A.); aiba@ncvc.go.jp (T.A.); tnoguchi@ncvc.go.jp (T.N.); kusanokengo@ncvc.go.jp (K.K.); hiyokoya@hctv.ne.jp (H.Y.); yasuda.satoshi.hp@mail.ncvc.go.jp (S.Y.); ogawah@ncvc.go.jp (H.O.); 3 Department of Statistics and Data Analysis, Center for Cerebral and Cardiovascular Disease Information, National Cerebral and Cardiovascular Center, Osaka 5648565, Japan; nakai.michikazu@ncvc.go.jp (M.N.); knishimu@ncvc.go.jp (K.N.)

**Keywords:** acute heart failure, hospital-acquired pneumonia, prognosis

## Abstract

The prognostic impact of hospital-acquired pneumonia (HAP) in acute heart failure (AHF) patients have not been fully elucidated. We evaluated 776 consecutive hospitalized AHF patients. The primary in-hospital outcomes were all-cause death and worsening heart failure (WHF), while the outcome following discharge was all-cause death. The clinical diagnosis of HAP was based on clinical practice guidelines by the Infectious Diseases Society of America and the American Thoracic Society. Patients with HAP had a significantly higher incidence of in-hospital death (12% vs. 1%, *p* < 0.001), WHF during the hospitalization (28% vs. 7%, *p* < 0.001), and longer length of hospital stay (*p* = 0.003) than those without. Among patients who survived at discharge, during a median follow-up period of 741 (interquartile range 422–1000) days, the incidence of all-cause death was significantly higher in patients with HAP than in those without (*p* < 0.001). In the multivariable Cox regression, HAP development was independently associated with all-cause death after discharge (HR [hazard ratio] 1.86, 95%CI [confidence interval] 1.08–3.19). Furthermore, older age (OR [odds ratio] 1.04, 95%CI 1.01–1.08), male sex (OR 2.21, 95%CI 1.14–4.28), and higher serum white blood cell count (OR 1.18, 95%CI 1.09–1.29) and serum C-reactive protein (OR 1.08, 95%CI 1.01–1.06) were independently associated with HAP development. In hospitalized patients with AHF, HAP development was associated with worse clinical outcomes, suggesting the importance of prevention and early screening for HAP.

## 1. Introduction

Acute heart failure (AHF) is a severe medical condition that requires intensive medical support, and most patients with AHF are elderly and at a high risk of hospital-acquired pneumonia (HAP) [1]. The increase in the prevalence and incidence of heart failure (HF) due to super-aging societies is a major growing public health concern worldwide [2], reflected in the high morbidity and mortality rates [3]. Moreover, HAP development in patients with AHF not only leads to worse clinical outcomes but also heavy economic burden [4].

HAP is a common infection that affects many hospitalized patients and has an unfavorable prognosis, particularly among older hospitalized patients or those with multiple comorbidities [5]. HAP is defined as an inflammatory condition of the lung parenchyma caused by infectious agents not present at least 48 hours or more after admission and does not include those intubated at the time of admission. Approximately half of patients with HAP have serious complications such as respiratory failure, septic shock, and/or acute kidney injury [6]. As patients often need additional care for these complications, the development of HAP is associated with longer length of hospital stay [7].

A recent study demonstrated that pneumonia was associated with increased short- and long-term risks of new-onset cardiovascular disease (CVD) [8]. However, the incidence, determinants, and prognostic implications of HAP in hospitalized patients with AHF have not been well investigated. Moreover, early identification of patients at a higher risk of HAP is important for risk stratification and preventive management in hospitalized patients with AHF. Therefore, the purpose of this study was first to investigate the incidence and the impact of HAP on short- and long-term outcomes, and second to evaluate which factors were independently associated with HAP development in hospitalized patients with AHF.

## 2. Methods

### 2.1. Study Design

Data from the National Cerebral and Cardiovascular Center Acute Decompensated Heart Failure (NaDEF) registry, which were obtained between January 2013 and May 2016 at the National Cerebral and Cardiovascular Center, Suita, Japan, were retrospectively analyzed. Details of the NaDEF registry have been described previously [9]. Briefly, the NaDEF registry is a single-center, observational cohort that includes all patients who require hospitalization with a diagnosis of AHF according to the Framingham criteria [10]. The study protocol of this registry was approved by the Institutional Review Board of the National Cerebral and Cardiovascular Center (M22-025 and M29-059) and was registered under the Japanese University Hospital Medical Information Network Clinical Trials Registration (UMIN000017024).

Acute coronary syndrome was defined based on ACCF (American College of Cardiology Foundation)/AHA (American Heart Association) guideline for the management of STEMI (ST-segment-elevation myocardial infarction) [11]. Follow-up was performed at 3, 6, 12, and 24 months after discharge by direct contact with patients or patients’ physicians at the hospital or outpatient clinic, telephone interview of patients, or, if deceased, of family members, and by mail, by dedicated coordinators and investigators which was described our previously study [9].

### 2.2. Blood Sampling Measurement

Venous blood samples were obtained for measurements of routine laboratory parameters on admission. The Controlling Nutritional Status (CONUT) score was calculated by the levels of serum albumin, the total peripheral lymphocyte count, and the total cholesterol concentration, which was found to be useful for risk stratification for AHF patients as well as assessing nutrition status [12]. The CONUT score was defined as the sum of these points based on a previous report that used preoperative serum samples [13].

### 2.3. Definition of HAP, Worsening Heart Failure and Worsening Renal Failure

The clinical diagnosis of HAP was based on the updated guidelines of the Infectious Diseases Society of America and the American Thoracic Society [14]. In brief, the diagnostic criteria included those with abnormal X-ray images indicating shadows in the lungs and the presence of at least two of the following: (i) fever ≥ 38 °C, (ii) white blood cell abnormalities (increase or decrease), and (iii) purulent secretions. HAP was recognized if patients developed pneumonia at least 48 hours or more after admission and were not intubated at the time of admission. Of note, there were 26 diagnosed with pneumonia on admission or within 48 hours from admission. Moreover, 15 patients were intubated on admission. We did not include these subjects as HAP patients. Worsening HF (WHF) was defined as worsening symptoms and signs of HF requiring intensification of intravenous therapy (i.e., a dose increase or re-administration) including loop diuretics, vasodilators, and inotropes or initiation of mechanical support after stabilization with initial treatment during hospitalization as used in major AHF clinical trials [15,16]. We assured the HF condition on the basis of patient’s symptom, physical examination, or imaging such as echocardiography.

Worsening renal failure (WRF) was assessed based on the maximum increase in serum creatinine from admission to any time during hospitalization. We defined it as an increase in serum creatinine of ≥ 0.3 mg/dL, which has been widely used in most prior studies [17,18].

### 2.4. Clinical Outcomes

Clinical outcomes that developed during hospitalization and after discharge were assessed separately to evaluate the impact of HAP on short- and long-term outcomes. The primary in-hospital outcomes were all-cause death and WHF, while the outcomes following discharge were all-cause death. The secondary outcome was length of hospital stay.

### 2.5. Statistical Analyzes

Continuous variables were presented as means ± standard deviations when normally distributed, and as medians and interquartile ranges (IQRs) when non-normally distributed. Parameters were compared between groups with and without HAP using unpaired *t*-tests or Mann–Whitney U tests for continuous variables and by Chi-squared tests or Fisher’s exact test for dichotomous variables, when appropriate.

The long-term cumulative incidence of all-cause death was estimated using Kaplan–Meier curves, and a log-rank (Mantel-Cox) test was performed to assess differences across these two groups. The association between parameters and all-cause death was assessed by Cox proportional hazards regression analysis. Univariable factors with *p* < 0.10 were identified. These factors were entered into the multivariable model to assess HAP impact on all-cause death (Model 1), and LVEF (left ventricular ejection fraction) ≥ 50% (categorical value) was entered alternative to continuous LVEF value (Model 2). Moreover, stepwise selection with a *p*-value of 0.10 for backward elimination was used to select the best predictive model (Model 3). We evaluated discriminative ability of other multivariable models with Harrell’s c-statistics [19]. Multivariable logistic regression analysis was performed based on the variables achieving *p* < 0.10 in univariable logistic regression analysis to assess the determinants of WHF during hospitalization and HAP development. We selected the covariates referred to by the published risk factors for HAP development [20]. All tests were two tailed, and a value of *p* < 0.05 was considered statistically significant. All analyses were performed with STATA^®^ 15 (Stata Corp, College Station, TX, USA).

## 3. Results

### 3.1. Study Population and Baseline Characteristics

**Figure 1:**
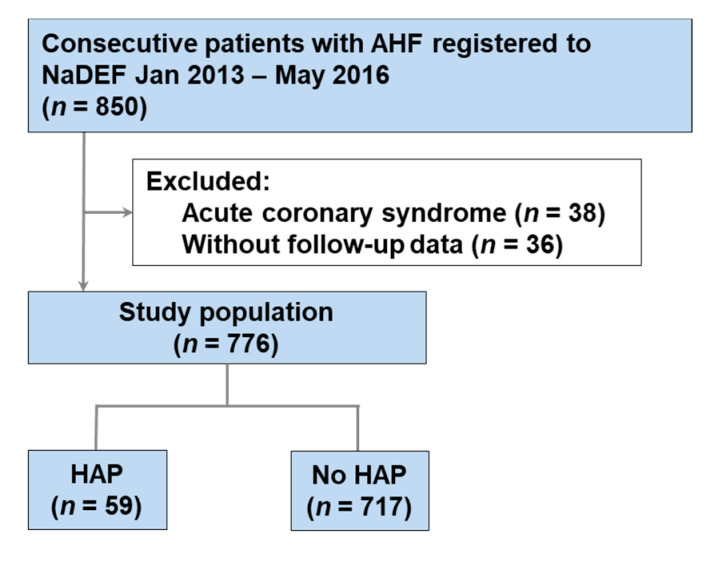
Flow diagram of the study. AHF—acute heart failure; NaDEF—National Cerebral and Cardiovascular Center Acute Decompensated Heart Failure; HAP—hospital-acquired pneumonia.

From the 850 consecutive patients with AHF enrolled in the NaDEF registry, those with acute coronary syndrome (*n* = 38) and those without follow-up data after discharge (*n* = 36) were excluded. Ultimately, 776 patients were examined. A study diagram is shown in Figure 1.

HAP developed in 59 (8%) patients during hospitalization. Baseline characteristics of the total 776 studied patients are presented in Table 1. Patients with HAP were older in age, predominantly male sex, and had a higher rate of intravenous loop diuretics use, higher serum creatinine, white blood cell (WBC) count, C-reactive protein (CRP), D-dimer, higher CONUT score, and lower serum albumin levels than those without HAP. Moreover, the prevalence of cardiovascular intensive care unit (ICU) admission was significantly higher in patients with HAP than in patients without HAP. There were no significant differences between the two groups in terms of body mass index, New York Heart Association functional class, LVEF, past history, etiology of HF, systolic blood pressure, plasma brain natriuretic peptide (BNP), estimated glomerular filtration rate, blood glucose levels, oral medication before admission, and worsening renal failure during hospitalization.

### 3.2. Clinical Outcomes During Hospitalization

**Figure 2:**
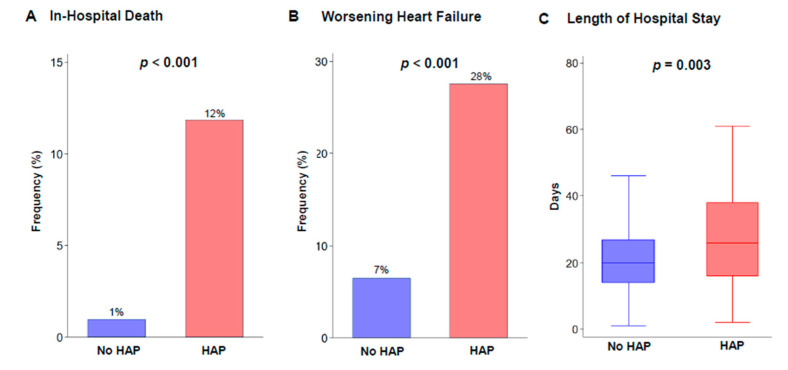
Incidence of adverse events during hospitalization. (**A**) In-hospital death, (**B**) Worsening heart failure, (**C**) Length of hospital stay. HAP, hospital-acquired pneumonia.

Clinical outcomes during hospitalization are shown in Figure 2. Overall, 60 patients experienced WHF during hospitalization and a total of 14 all-cause deaths occurred. Patients with HAP had a significantly higher incidence of in-hospital death and WHF during hospitalization than those without HAP. Moreover, in a multivariable logistic regression, HAP was independently associated with WHF during hospitalization (Table 2). In addition, patients with HAP had a longer length of hospital stay than those without HAP.

### 3.3. Long-Term Clinical Outcomes after Discharge and Determinants of HAP

**Figure 3:**
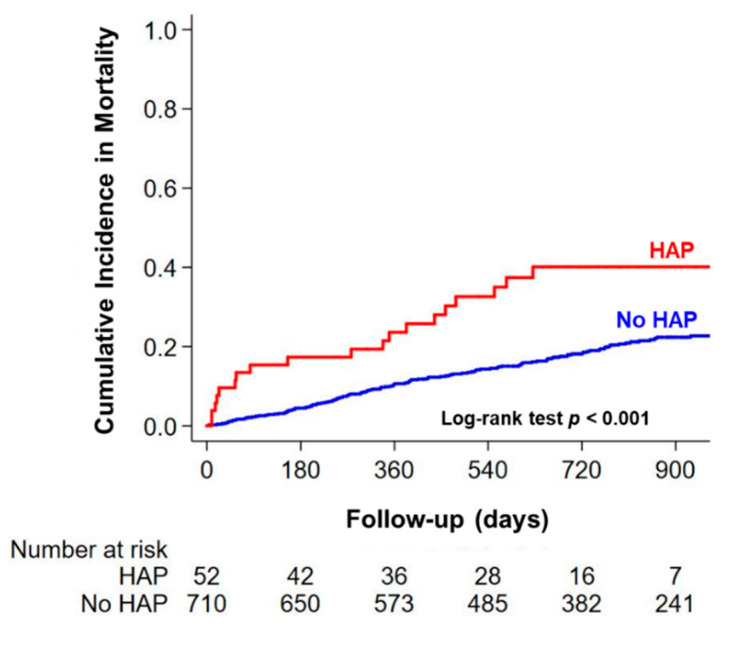
Kaplan-Meier analysis of all-cause death after discharge in patients with or without HAP, hospital-acquired pneumonia.

Among patients who survived at discharge, during a median follow-up period of 741 (IQR 422–1000) days, all-cause death occurred in 152 (20%) study patients. The incidence of all-cause death was significantly higher in patients with HAP than in those without HAP (Figure 3). In the multivariable Cox regression analyzes, HAP development was independently associated with all-cause death after discharge even after adjustment for powerful prognostic AHF variables (Table 3). Interaction analysis revealed that there was no significant interaction on plasma BNP levels and LVEF for the prognostic value of HAP (*p* for interaction = 0.50, 0.88, respectively).

Regarding determinants of HAP, in a multivariable logistic regression, older age, male sex, serum WBC count, and serum CRP level on admission were independently associated with HAP development (Table 4).

## 4. Discussion

First, patients who developed HAP had a higher incidence of in-hospital death and WHF during hospitalization and a longer length of hospital stay than those who did not develop HAP. Second, HAP development was independently associated with all-cause mortality after discharge even after adjustment for powerful prognostic variables. Finally, older age, male sex, serum WBC count, and serum CRP levels on admission were independently associated with HAP development in hospitalized patients with AHF. These findings indicate the importance of HAP development for further risk stratification in hospitalized patients with AHF.

Although the prevalence of HAP ranges from 0.5% to more than 2% following hospital admission [14,21], the prevalence of HAP in hospitalized patients with AHF is uncertain. Our study indicated that the prevalence of HAP was approximately 8% in hospitalized patients with AHF, which was relatively higher than that of other diseases that required hospitalization. Generally, mechanical ventilation performed for more than 48 hours, residence in an ICU, length of hospital stay, underlying illness severity, and presence of comorbidities are considered major risk factors for HAP [22]. AHF is defined as a new onset of severe HF or the sudden intensification of chronic HF, which are life-threating conditions that require hospitalization. Various manifestations of these conditions such as respiratory failure and cardiogenic shock often require ICU admission for mechanical ventilation or mechanical circulatory support [23]. Therefore, these patients appear to be at a very high risk of HAP. Older age, male sex, and the pro-inflammatory state were independent determinants of HAP development in our AHF cohort, and these factors are known to be associated with frailty. Especially in frail patients, aspiration pneumonia due to impaired swallowing function is frequently observed [24], These findings indicate that physicians should take special care in monitoring patients with AHF who have these characteristics to prevent HAP.

In the present study, we also demonstrated that patients who developed HAP had worse short-term outcomes, such as a higher incidence of WHF and in-hospital death during hospitalization, than those who did not develop HAP. Sepsis is characterized by an increase in oxygen demand related to severe infection inflammatory response, known to be generated by an inappropriate immune response [25]. Pneumonia is one of the most common causes of severe sepsis, accounting for nearly one-half of cases reported [26]. Although the mechanism of cardiovascular system impairment during sepsis has not been fully elucidated, acute infections can adversely affect the systemic circulation, impair cardiac function, reduce systemic vascular resistance, increase oxygen consumption, and cause tachycardia, leading to an increase in workload for the heart [27]. Acute infections also promote inflammatory activity in coronary atherosclerotic plaques and induce prothrombotic changes in the blood and endothelium that result in plaque instability and coronary thrombosis facilitation, which can promote myocardial ischemia [28]. These reactions are often more severe in hospitalized patients with AHF and can lead to WHF resulting in prolonged length of hospital stay; the reactions can also increase the incidence of in-hospital death. Notably, the presence of cardiovascular dysfunction in patients with sepsis is associated with a significantly increased mortality rate of 70% to 90% compared to 20% in those without sepsis [29].

Interestingly, HAP development was also associated with worse long-term survival outcomes even after adjusting for the powerful prognostic factors for AHF. The exact reason for this finding is unclear; however, we can speculate as follows. First, persistent inflammation after pneumonia can contribute to subsequent CVD progression [8,30]. One prospective cohort study has shown that patients with pneumonia who left the hospital with higher interleukin-6 levels at discharge were associated with increased subsequent mortality despite clinical recovery [31]. Second, higher levels of coagulation markers are also commonly observed at the time of hospital discharge in patients who have recovered from pneumonia. One study showed that higher D-dimer and thrombin-antithrombin complex levels were associated with a higher risk of subsequent death, particularly due to CVD [32]. These pro-inflammatory and hypercoagulable states may increase the risk of cardiovascular events including myocardial infarction or cerebral infarction [33]. Finally, acute organ injury such as acute lung or kidney injury due to pneumonia during hospitalization might progress to organizing pneumonia or chronic kidney disease after discharge, leading to worse long-term clinical outcomes.

Our study has several clinical implications. We found that HAP occurs at a relatively high rate in patients with AHF, which can cause serious problems. Moreover, patients who demonstrated frailty were found to be at a high risk of HAP. According to a prospective observational study of 4.4 million hospitalized patients, multicomponent interventions reduced HAP development and associated mortality [34]. Among the various preventive interventions, oral care is the most effective method to reduce HAP development [35]. In addition, early screening for dysphagia in patients who experience a stroke has been reported to reduce the incidence of pneumonia and shorten the length of hospital stay [36]. Therefore, it is important to perform swallowing assessments and rehabilitation in patients with a history of frailty or cerebral infarctions as early HAP screening. Taken together, we believe that the early screening and preventive interventions for patients with AHF at a high risk of HAP would help to improve outcomes. Moreover, the prior HF hospitalizations was also independently associated with long-term mortality, which was consist with the previous study [37]. Special attention should be paid for these patients.

There are potential limitations of the present study which should be acknowledged. First, the sample size was relatively small and from a single center, thereby limiting the ability to generalize the findings and the statistical power for detecting differences in negative data; however discriminative ability of multivariable models was modestly sufficient. Therefore, further multi-center prospective studies with a larger population are warranted. Second, some information that may have affected the outcomes and incidence of HAP was unavailable or incomplete, such as time from admission to onset of pneumonia, the hospital antibiograms, and types of specific type of antibiotic therapy. Third, we had no data regarding some inflammatory markers (e.g., procalcitonin) and duration of antibiotics and diuretic treatments. Forth, we found an association between development of HAP and WHF during hospitalization in logistic regression, but this finding would provide only associative evidence, not causative evidence. Fifth, there was no data regarding HAP development after discharge. Finally, although the clinical diagnosis of HAP was based on major guidelines, patients with infections other than pneumonia who had abnormal X-ray shadows due to HF congestion may have been misdiagnosed with pneumonia. In these cases, we assessed abnormal X-ray images as a change in radiographic appearance from previously available imaging. Moreover, when we could not get the data on the quality of secretions, we diagnosed those as HAP who met all the other two findings (i. fever, ii. white blood cell abnormalities).

## 5. Conclusions

Our analyses revealed that patients with AHF who developed HAP as well as the prior HF hospitalization had unfavorable long-term mortality. Moreover, AHF patients with HAP during the hospitalization had a higher incidence of in-hospital death, longer length of hospital stay, and WHF during hospitalization. It would be necessary to give special attention on prevention of HAP for these patients.

## Figures and Tables

**Table 1:** Baseline characteristics.

Variable	Overall	HAP	No HAP	*p*-Value
Number	776	59	717	-
Age, year	75 ± 12	79 ± 9	75 ± 12	0.011
Male, *n* (%)	467 (60)	44 (75)	423 (59)	0.019
Body mass index, kg/m^2^	22.7 (20.3−25.4)	21.9 (19.45−24.25)	22.8 (20.4−25.5)	0.109
NYHA class III or IV, *n* (%)	592 (89)	45 (96)	574 (89)	0.138
Ischemic etiology, *n* (%)	185 (24)	20 (34)	165 (23)	0.059
LVEF, %	38 ± 17	41 ± 18	38 ± 17	0.24
Past History, *n* (%)				
Hypertension	545 (74)	50 (85)	495 (73)	0.051
Diabetes	266 (36)	18 (31)	248 (37)	0.34
HF admission	333 (45)	28 (47)	305 (45)	0.72
Atrial arrhythmia	386 (52)	36 (61)	350 (52)	0.169
Cerebrovascular disease	196 (27)	19 (32)	177 (26)	0.32
Malignancy	111 (15)	11 (19)	100 (15)	0.42
Chronic kidney disease	378 (52)	32 (54)	346 (51)	0.67
COPD	33 (4)	5 (8)	28 (4)	0.123
Etiology, *n* (%)				
ICM	185 (24)	20 (34)	165 (23)	0.059
DCM	81 (10)	3 (5)	78 (11)	0.162
HHD	182 (24)	10 (17)	172 (24)	0.22
Other	328 (42)	26 (44)	302 (42)	0.77
Current smoking, *n* (%)	77 (19)	4 (11)	73 (20)	0.21
Habitual drinking, *n* (%)	164 (47)	12 (44)	152 (48)	0.76
Systolic BP on admission, mm Hg	140 ± 32	139 ± 32	140 ± 32	0.90
Heart rate on admission, beat/min	92 ± 28	96 ± 28	91 ± 28	0.25
Laboratory Data on Admission				
White blood cell count, 10^3^/μL	6.4 (5.1−8.4)	8.3 (6.0−12.4)	6.4 (5.1−8.1)	<0.001
CRP, mg/dL	0.42 (0.14−1.41)	2.18 (0.65−6.17)	0.38 (0.13−1.14)	<0.001
Serum albumin, g/dL	3.8 ± 0.4	3.6 ± 0.5	3.8 ± 0.4	0.004
Total bilirubin, mg/dL	0.7 (0.5−1.1)	0.7 (0.4−1.0)	0.7 (0.5−1.1)	0.159
Sodium, mEq/L	139.5 ± 4.2	139.3 ± 4.5	139.6 ± 4.2	0.65
BNP, pg/mL	600 (323−1116)	736 (230−1395)	595 (326−1092)	0.63
Troponin T, ng/mL	0.04 (0.02−0.07)	0.04 (0.03−0.09)	0.04 (0.02−0.07)	0.051
Creatinine, mg/dL	1.1 (0.9−1.6)	1.2 (0.9−1.9)	1.1 (0.9−1.5)	0.042
BUN, mg/dL	23 (17−33)	25 (18−41)	23 (17−32)	0.093
eGFR, mL/min/1.73m^2^	60.0 (39.2−78.2)	50.0 (34.7−72.1)	60.8 (40.3−78.4)	0.100
Hemoglobin, g/dL	12.0 ± 2.1	11.4 ± 2.3	12.1 ± 2.1	0.033
Blood glucose, mg/dL	126 (106−166)	130 (103−195)	126 (106−164)	0.50
HbA1c, %	6.2 ± 1.0	6.2 ± 1.0	6.2 ± 1.0	0.59
D-dimer, mg/dL	2.0 (1.1−4.0)	3.0 (1.4−7.1)	2.0 (1.1−3.9)	0.009
CONUT score	2.0 (2.0−4.0)	4.0 (2.0−6.0)	2.0 (2.0−4.0)	<0.001
Medications before Admission, *n* (%)				
ACE inhibitors/ARBs	384 (49)	28 (47)	356 (50)	0.75
Beta-blockers	383 (49)	26 (44)	357 (50)	0.40
Diuretics	414 (53)	33 (56)	381 (53)	0.68
Spironolactone	149 (19)	15 (25)	134 (19)	0.21
Medications at Discharge, *n* (%)				
ACE inhibitors/ARBs	512 (71)	33 (63)	479 (71)	0.22
Beta-blockers	520 (72)	32 (62)	488 (73)	0.077
Diuretics	598 (83)	41 (79)	557 (83)	0.43
Spironolactone	285 (40)	17 (33)	268 (40)	0.34
Intravenous Treatments, *n* (%)				
Loop diuretics	554 (71)	49 (83)	505 (70)	0.039
Vasodilators	627 (81)	52 (88)	575 (80)	0.137
Inotropes	103 (13)	7 (12)	96 (13)	0.74
Dose of intravenous loop diuretics, mg/day	20 (20−40)	20 (20−40)	20 (20−40)	0.186
CICU admission, n (%)	177 (23)	24 (41)	153 (21)	0.001
WRF during hospitalization, n (%)	325 (42)	33 (50)	292 (42)	0.190

Values are mean ± standard deviation, median (interquartile range) or percentages. ACE, angiotensin converting enzyme; ARBs, angiotensin II receptor blockers; BNP, brain natriuretic peptide; BP, blood pressure; BUN, blood urea nitrogen; CICU, cardiovascular intensive care unit; CONUT, controlling nutritional status; COPD, chronic obstructive pulmonary disease; CRP, C-Reactive Protein; DCM, dilated cardiomyopathy; eGFR, estimated glomerular filtration rate; HAP, hospital acquired pneumonia; HbA1c, hemoglobin A1c; HF, heart failure; HHD, hypertensive heart disease; ICM, ischemic cardiomyopathy; LVEF, left ventricular ejection fraction; NYHA, New York Heart Association; WRF, Worsening renal failure.

**Table 2:** Determinants of worsening heart failure during hospitalization.

Variable	Univariable Analysis		Multivariable Analysis	
	OR (95%CI)	*p*-Value	OR (95%CI)	*p*-Value
HAP	5.69 (2.97−10.9)	<0.001	4.91 (2.44−9.89)	<0.001
eGFR, mL/min/1.73 m^2^	0.96 (0.96−0.98)	<0.001	0.98 (0.96−0.99)	0.004
Systolic BP, 10 mm Hg	0.85 (0.77−0.93)	0.001	0.88 (0.79−0.97)	0.011
Log BNP, pg/mL	1.55 (1.15−2.10)	0.004	1.28 (0.94−1.74)	0.122
Serum sodium, mEq/L	0.94 (0.88−0.99)	0.018	0.96 (0.91−1.02)	0.39
LVEF, %	0.99 (0.98−1.01)	0.28	Not selected	-
History of cerebrovascular disease	0.59 (0.30−1.17)	0.130	Not selected	-
Serum albumin, 0.2 g/dL	0.91 (0.81−1.02)	0.108	Not selected	-
NYHA class III or IV	1.31 (0.84−2.06)	0.23	Not selected	-
Age, year	0.99 (0.97−1.01)	0.51	Not selected	-
Sex, male	0.92 (0.54−1.57)	0.76	Not selected	-
Body mass index, kg/m^2^	0.98 (0.92−1.04)	0.49	Not selected	-

CI, confidence interval; OR, odds ratio. Other abbreviations as in Table 1. All data were obtained on admission.

**Table 3:** Cox proportional hazards model for determinants of all-cause death after discharge.

Variables	Univariable Analysis		Multivariable Analysis: Model 1		Multivariable Analysis: Model 2		Multivariable Analysis: Model 3	
	HR (95%CI)	*p*-Value	HR (95%CI)	*p*-Value	HR (95%CI)	*p*-Value	HR (95%CI)	*p*-Value
HAP	2.45 (1.51−3.97)	<0.001	1.86 (1.08−3.19)	0.025	1.47 (0.77−2.79)	0.024	2.14 (1.31−3.49)	0.002
Serum albumin, 0.2 g/dL	0.78 (0.73−0.84)	<0.001	0.82 (0.76−0.89)	<0.001	0.80 (0.74−0.88)	<0.001	0.79 (0.74−0.85)	<0.001
Prior HF admission	2.90 (2.05−4.10)	<0.001	2.04 (1.40−2.97)	<0.001	1.76 (1.17−2.65)	0.007	2.41 (1.69−3.43)	<0.001
eGFR, mL/min/1.73 m^2^	0.98 (0.98−0.99)	<0.001	0.99 (0.98−0.997)	0.006	0.99 (0.98−0.99)	<0.001	0.99 (0.98−0.994)	<0.001
Serum sodium, mEq/L	0.93 (0.90−0.97)	<0.001	0.96 (0.92−0.997)	0.033	0.96 (0.92−0.998)	0.0042	0.96 (0.93−0.99)	0.024
Use of Loop diuretics	0.78 (0.56−1.09)	0.142	Not selected	-	Not selected	-	0.73 (0.51−1.03)	0.077
Heart rate, 10 beat/min	0.92 (0.86−0.97)	0.006	0.99 (0.92−1.06)	0.70	0.97 (0.89−1.05)	0.48	Not selected	-
Body mass index, kg/m^2^	0.88 (0.84−0.92)	<0.001	0.92 (0.88−0.97)	0.002	0.92 (0.87−0.97)	0.004	Not selected	-
Systolic BP, 10 mmHg	0.90 (0.85−0.96)	<0.001	0.97 (0.91−1.03)	0.31	0.99 (0.93−1.06)	0.80	Not selected	-
Age, year	1.03 (1.02−1.05)	<0.001	1.02 (0.997−1.04)	0.096	1.01 (0.99−1.03)	0.45	Not selected	-
Log BNP, pg/mL	1.38 (1.16−1.65)	<0.001	1.07 (0.88−1.29)	0.49	1.03 (0.83−1.29)	0.76	Not selected	-
History of COPD	2.31 (1.31−4.09)	0.004	1.69 (0.92−3.10)	0.090	1.82 (0.90−3.66)	0.093	Not selected	-
Hemoglobin, g/dL	0.83 (0.76−0.89)	<0.001	0.996 (0.91−1.09)	0.93	0.98 (0.89−1.09)	0.72	Not selected	-
CRP, mg/dL	1.06 (1.02−1.10)	0.004	1.00 (0.96−1.05)	0.89	1.00 (0.94−1.05)	0.88	Not selected	-
LVEF ≥ 50%	0.85 (0.58−1.24)	0.40	Not selected	-	0.85 (0.54−1.35)	0.49	Not selected	-
LVEF, %	1.00 (0.99−1.01)	0.80	Not selected	-	Not selected	-	Not selected	-
WBC, 10^3^/μL	0.96 (0.90−1.02)	0.157	Not selected	-	Not selected	-	Not selected	-
ICM	0.87 (0.59−1.27)	0.47	Not selected	-	Not selected	-	Not selected	-
DCM	0.90 (0.69−1.17)	0.44	Not selected	-	Not selected	-	Not selected	-
HHD	1.09 (0.97−1.26)	0.153	Not selected	-	Not selected	-	Not selected	-
NYHA class III or IV	1.26 (0.71−2.24)	0.43	Not selected	-	Not selected	-	Not selected	-
Sex, male	1.20 (0.86−1.67)	0.29	Not selected	-	Not selected	-	Not selected	-

CI, confidence interval; HR, hazard ratio. Other abbreviations as in Table 1. All data were obtained on admission. Model 1: significant covariates were identified in univariable analysis. Model 2: addition of LVEF ≥ 50% (categorical value) to Model 1. Model 3: stepwise backward selection. Harrel’s c-index of Model 1, Model 2, and Model 3 are 0.76, 0.76, and 0.74, respectively.

**Table 4:** Determinants of development of HAP.

Variable	Univariable Analysis		Multivariable Analysis	
	OR (95%CI)	*p*-Value	OR (95%CI)	*p*-Value
Age, year	1.04 (1.01−1.06)	0.011	1.04 (1.01−1.08)	0.006
Sex, male	2.04 (1.11−3.73)	0.021	2.21 (1.14−4.28)	0.019
WBC, 10^3^/μL	1.20 (1.121.29)	<0.001	1.18 (1.09−1.29)	<0.001
CRP, mg/dL	1.15 (1.09−1.21)	<0.001	1.08 (1.01−1.16)	0.017
Serum albumin, 0.2 g/dL	0.84 (0.75−0.95)	0.004	0.95 (0.83−1.09)	0.45
Creatinine, mg/dL	1.16 (0.995−1.36)	0.051	1.08 (0.88−1.33)	0.46
Hemoglobin, g/dL	0.87 (0.77−0.99)	0.033	0.93 (0.80−1.08)	0.34
D-dimer, mg/dL	1.01 (0.99−1.02)	0.33	Not selected	-
Body mass index, kg/m^2^	0.97 (0.90−1.03)	0.32	Not selected	-
History of COPD	2.14 (0.80−5.77)	0.132	Not selected	-
History of diabetes	0.76 (0.43−1.35)	0.35	Not selected	-
History of cerebrovascular disease	1.33 (0.75−2.37)	0.32	Not selected	-
Systolic BP, 10 mm Hg	0.99 (0.91−1.08)	0.90	Not selected	-
Log BNP, pg/mL	1.03 (0.78−1.37)	0.81	Not selected	-
LVEF, %	1.01 (0.99−1.03)	0.24	Not selected	-

CI, confidence interval; OR, odds ratio. Other abbreviations as in Table 1.
